# SinglePass Kronos electrocautery device for closure of liver mass image-guided biopsy: a series of five case reports

**DOI:** 10.3389/fonc.2026.1766048

**Published:** 2026-04-20

**Authors:** Tust Techasith, Abhishek Jairam, Thomas Velling, Avinash Mesipam, Christopher Baker, Trushar Patel, Behroz Oftadeh, Vincent Nguyen, Quan Dang, Lindsey Young, Alexander S. Misono

**Affiliations:** Hoag Memorial Hospital Presbyterian, Newport Radiology, Newport Beach, CA, United States

**Keywords:** bleeding, case report, core needle biopsy, hemostasis, image-guided biopsy, liver mass biopsy, percutaneous liver mass biopsy

## Abstract

Percutaneous liver mass biopsies, including coaxial needle biopsy (CNB), have become the preferred method for obtaining tissue samples in diagnosing or characterizing liver masses. Despite the advent of intraprocedural computed tomography (CT) and/or ultrasound image guidance as well as various biopsy tract “plugging” techniques, bleeding remains a frequent and potentially highly morbid complication. As such, patients often undergo extended monitoring. Furthermore, in cases of bleeding, patients may require additional imaging, intervention, and even surgery. Death, while very rare, is known to occur. We report a retrospective, observational, non-comparative series of five image-guided liver mass biopsy cases in which hemostasis and biopsy tract closure were obtained with a new electrocautery hemostasis device called the SinglePass Kronos electrocautery device. All biopsy procedures were technically successful, and there were no reported complications out to 30 days. Post-procedure imaging confirmed the absence of bleeding in all five cases. The SinglePass electrocautery device is a potentially valuable tool in minimizing bleeding complications in percutaneous solid organ biopsy procedures and has particular utility in high-risk biopsies such as liver mass biopsies. Larger-scale studies of this new device may be warranted.

## Introduction

Image-guided coaxial needle biopsy (CNB) is a favored technique for obtaining high-yield solid organ tissue samples percutaneously. CNB enables minimally invasive diagnosis of various solid organ disorders without the need for surgery and is commonplace in the management and workup of various diseases, including the investigation of liver masses (1, 2). However, percutaneous CNB procedures can also result in adverse events related to needle passes and capsular organ punctures during tissue collection. This may be further accentuated in high-risk patients with inherent coagulopathies, are on anticoagulation medication, or often a combination of the two (3–5). As such, both minor bleeding and severe hemorrhage, including bleeding requiring transfusion or intervention to stop bleeding, while typically rare, have been reported with event rates of up to 7.4% (6–8). In addition, it is difficult to predict and detect which patients may bleed or develop bleeding complications after CNB procedures. As such, there has been increasing use of various techniques and agents amongst interventional radiologists to plug biopsy tracts and promote hemostasis (5, 7, 9, 10).

Despite these methods, patients continue to require bedrest and close monitoring in the post-procedural setting, often for many hours. Some practices employ manual pressure and pressure bandages. Serious hemorrhage requiring blood transfusions, endovascular intervention such as angiography and embolization, or surgical repair can become required; patient mortality is also known to occur (1, 3, 6, 11). The SinglePass Kronos electrocautery device was developed to address these common and potentially serious complications of percutaneous CNB.

We previously reported two case series in medical liver (12) and medical kidney (13) CNB using the SinglePass electrocautery device following its introduction onto the market in the United States. In this case series, we introduce an initial experience of 5 new cases of liver mass CNB.

## Materials and methods

Ultrasound- or CT-guided, percutaneous, liver mass CNB was performed in both inpatient and outpatient settings for various liver mass tissue assessments in five patients. Patients were assessed for relative and absolute procedural contraindications, including the use of antiplatelet and/or anticoagulation medications, according to the Society of Interventional Radiology (SIR) guidelines.

**Figure 1:**
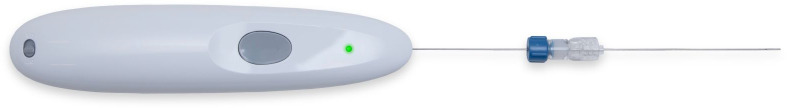
SinglePass electrocautery device.

At our institution, medical liver mass biopsies are routinely performed with either a 19/20-gauge or 17/18-gauge Bard Max-Core Disposable Core Biopsy Instruments (Becton Dickinson, Inc., Franklin Lakes, NJ). In these cases, 17/18-gauge biopsies were performed. Subsequently, the SinglePass electrocautery device (**Figure 1**, SinglePass, Inc., Lake Forest, CA) was utilized for hemostasis of the needle tract. The SinglePass device is a disposable, battery-operated electrocautery device utilized to control bleeding following CNB of solid organs (i.e., liver, kidney, lung, etc.). It consists of an ergonomic handle that activates a probe with an electrically activated tip that heats up to a maximum of 90˚C to cauterize tissue. Informed consent to perform the procedure was obtained from each patient. SinglePass device depth of travel was limited with a depth gauge that is adjusted to match the intended cautery depth, typically matching the biopsy device throw length (10–22 mm).

Following tissue sample acquisition, the SinglePass device was inserted into the introducer needle, and the depth gauge was luer-locked onto the needle. The device was activated and electrocautery was applied within the mass for approximately 10 seconds. The introducer needle and the SinglePass device were then slowly withdrawn as a unit with the device activated. Withdrawal speed was determined by operator preference, but total cautery time was typically 30 seconds. The device was then powered off and discarded.

Patients’ peri- and post-operative electronic medical record (EMR) documentation and applicable imaging studies were reviewed to identify any adverse events. Bleeding was adjudicated by the investigators using the SIR criteria (14). Patients with adverse events at this center tend to return to an in-network urgent care or emergency department, but each patient’s EMR was reviewed for CNB-related events through 30 days post-biopsy both within and outside of the network. No patient-reported data were obtained in this series.

## Results

### Case #1

**Figure 2:**
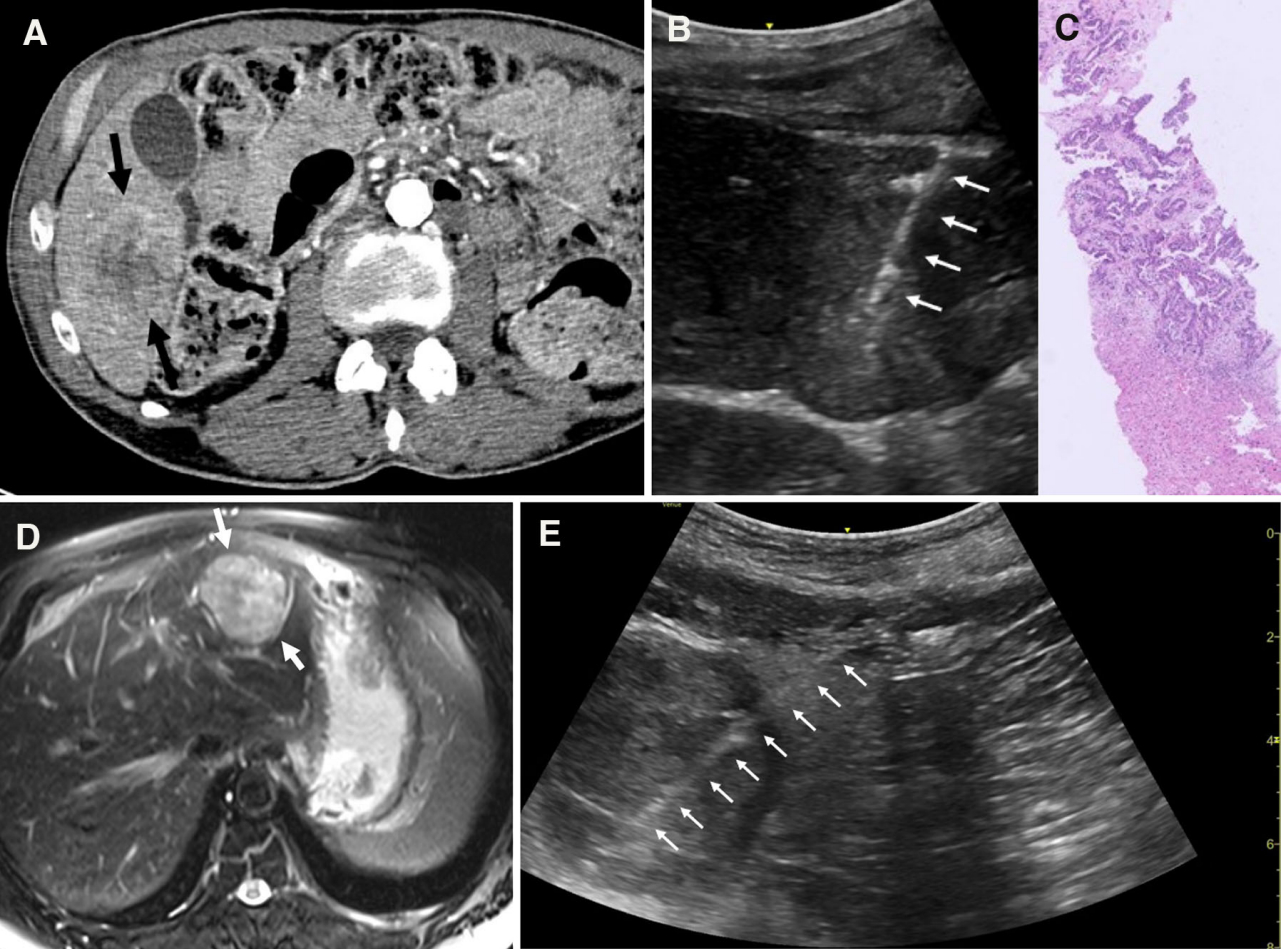
Cases #1 & 2, Panel **(A)** Case #1 liver mass seen by pre-biopsy CT imaging (black arrows). Panel **(B)** Case #1 cautery tract following biopsy (white arrows). Panel **(C)** Case #1 pathology reveals irregular, infiltrative glandular structures with hyperchromatic nuclei in keeping with adenocarcinoma of colorectal origin. These malignant units are encased within a desmoplastic stroma, demonstrating a clear fibrotic interface that compresses upon the adjacent benign hepatic parenchyma. Panel **(D)** Case #2 pre-procedure T2 fat-saturated axial MRI image shows liver mass (white arrows). Panel **(E)** Case #2 echogenic needle tract (white arrows) is demonstrated in ultrasound image post-biopsy and SinglePass device electrocautery (white arrows).

A 68-year-old male patient with a history of a rectal mass underwent outpatient CNB following identification of a liver mass in the right lobe of the liver on CT imaging (**Figure 2A**). He was not taking antiplatelet or anticoagulant medications, and his pre-procedure labs were unremarkable.

Four core samples were obtained under ultrasound guidance. The biopsy procedure, followed by SinglePass device electrocautery, was completed without complications and the patient was discharged 4 hours post-procedure. Immediately post-procedure ultrasound imaging confirmed absence of bleeding complications and a clearly defined cautery tract (**Figure 2B**).

Pathology resulted as adenocarcinoma in a background of extensive fibrosis in keeping with metastatic disease of colorectal origin (**Figure 2C**). There were no complications reported in the 30 days following the procedure. The patient underwent further workup and was found to have rectal cancer, for which he underwent chemotherapy and radiation, liver resection, and subsequent low anterior resection with diverting loop ileostomy. Most recently, he underwent ileostomy reversal and closure.

### Case #2

A 61-year-old male underwent an outpatient liver mass CNB after the incidental finding of a liver mass on magnetic resonance imaging (MRI) (**Figure 2C**). The patient reportedly had undergone a consumer screening test for several genetic and molecular markers and was reportedly told he had a liver abnormality, which ultimately led to the MRI examination. Past medical history was only notable for hepatic steatosis as a risk factor.

He was taking aspirin 81 mg/day, which was held for 5 days prior to the biopsy procedure as per SIR guidelines. His pre-procedure blood work was unremarkable.

Using ultrasound guidance, four core samples were obtained, followed by SinglePass device electrocautery. There were no complications during the procedure, and the patient was discharged 4 hours later. Ultrasound imaging confirmed no bleeding following the procedure with clear visualization of the cautery tract (**Figure 2D**).

Pathology was diagnostic for hepatocellular carcinoma. No complications were reported in the 30 days following the procedure. The patient underwent surgical resection followed by atezolizumab combined with bevacizumab and is currently being followed clinically, serologically, and by imaging.

### Case #3

**Figure 3:**
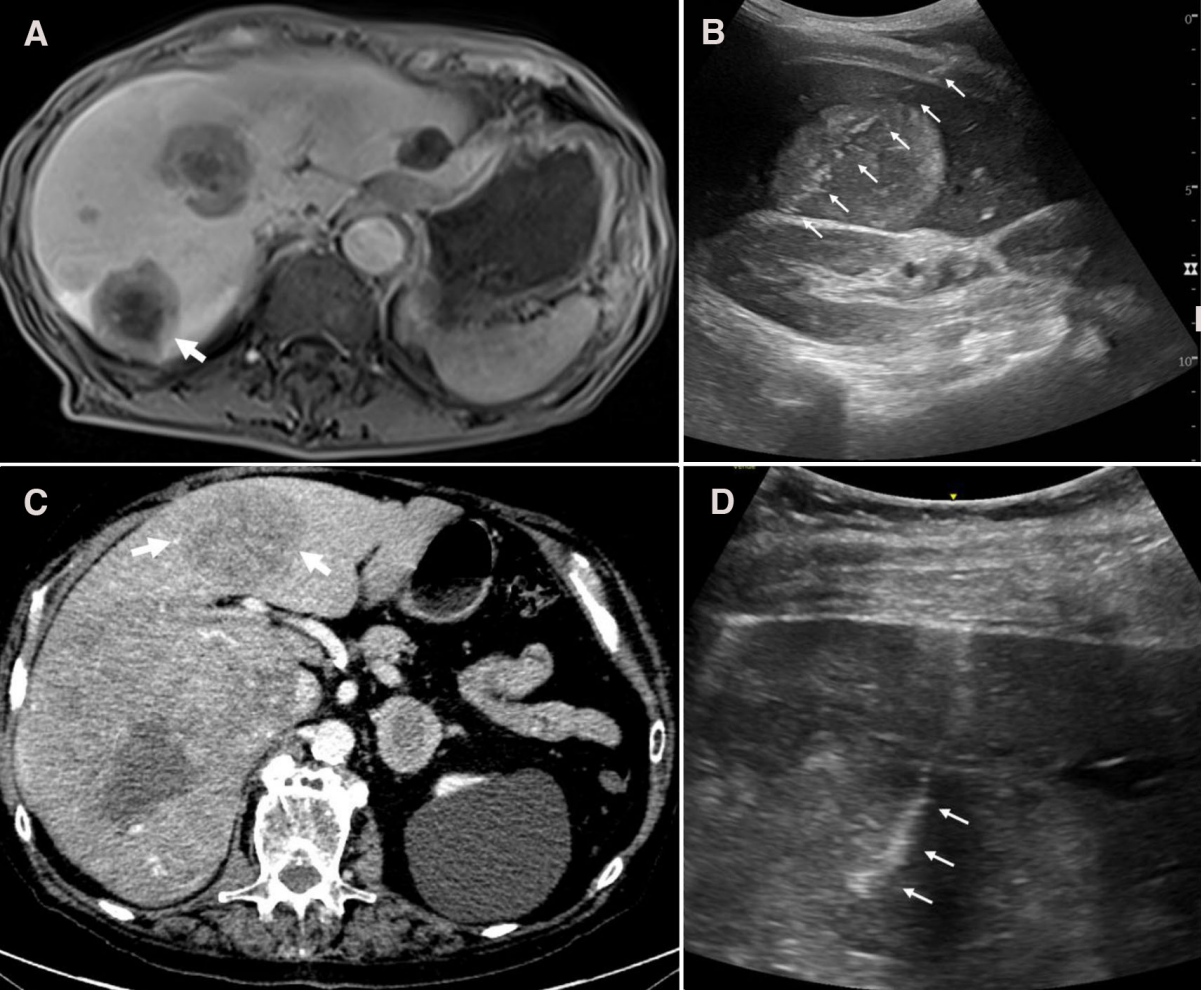
Cases #3 & 4, Panel **(A)** Case #3 pre-procedure post-contrast T1 MRI showing liver mass (white arrow). Panel **(B)** Case #3 post-biopsy and SinglePass device electrocautery needle path (white arrows). Panel **(C)** Case #4 pre-procedure contrast-enhanced CT imaging showing liver mass (white arrows). Panel **(D)** Case #4 post-biopsy and SinglePass device electrocautery ultrasound image showing needle tract (white arrows).

An 87-year-old male underwent an outpatient liver mass CNB due to liver masses discovered following an abdominal CT scan and subsequent MRI (**Figure 3A**). The patient had abdominal imaging because he was suffering from abdominal pain for several weeks. CT scan revealed masses and the MRI was obtained for further imaging characterization.

He was not taking any antiplatelet or anticoagulant medication and pre-procedure labs were normal. With ultrasound guidance, three core samples were obtained, followed by SinglePass device electrocautery. Post-procedure ultrasound imaging confirmed no bleeding (**Figure 3B**). The patient was discharged four hours following the procedure.

Pathology demonstrated metastatic adenocarcinoma suspicious for a gastrointestinal tract primary. There were no reported complications in the 30 days after the procedure. A colonoscopy was performed which revealed a large colonic mass at the hepatic flexure of colon. The patient was thereafter started on FOLFOX and bevacizumab.

### Case #4

An 82-year-old male with liver masses identified by contrast-enhanced CT imaging underwent a CNB during an inpatient stay (**Figure 3C**). The patient was noted to have abdominal pain and abnormal transaminases and was admitted for expedited workup.

The patient was taking rivaroxaban pre-procedure, which was held for 48 hours pre-procedure as per SIR guidelines. He also had a slightly elevated INR (1.3) in pre-biopsy blood work. An ultrasound-guided biopsy yielded four core samples and was immediately followed by SinglePass device electrocautery. There were no complications from the procedure and post-biopsy ultrasound imaging confirmed needle tract hemostasis (**Figure 3D**). The patient was kept on bedrest for four hours following the procedure and then resumed normal activity.

Pathology revealed moderately differentiated hepatocellular carcinoma. No complications were reported in the subsequent 30 days. The hepatocellular carcinoma was deemed unresectable, and the patient opted to enter a Phase 2 clinical study for treatment of unresectable and/or locally advanced or metastatic hepatocellular carcinoma.

### Case #5

**Figure 4:**
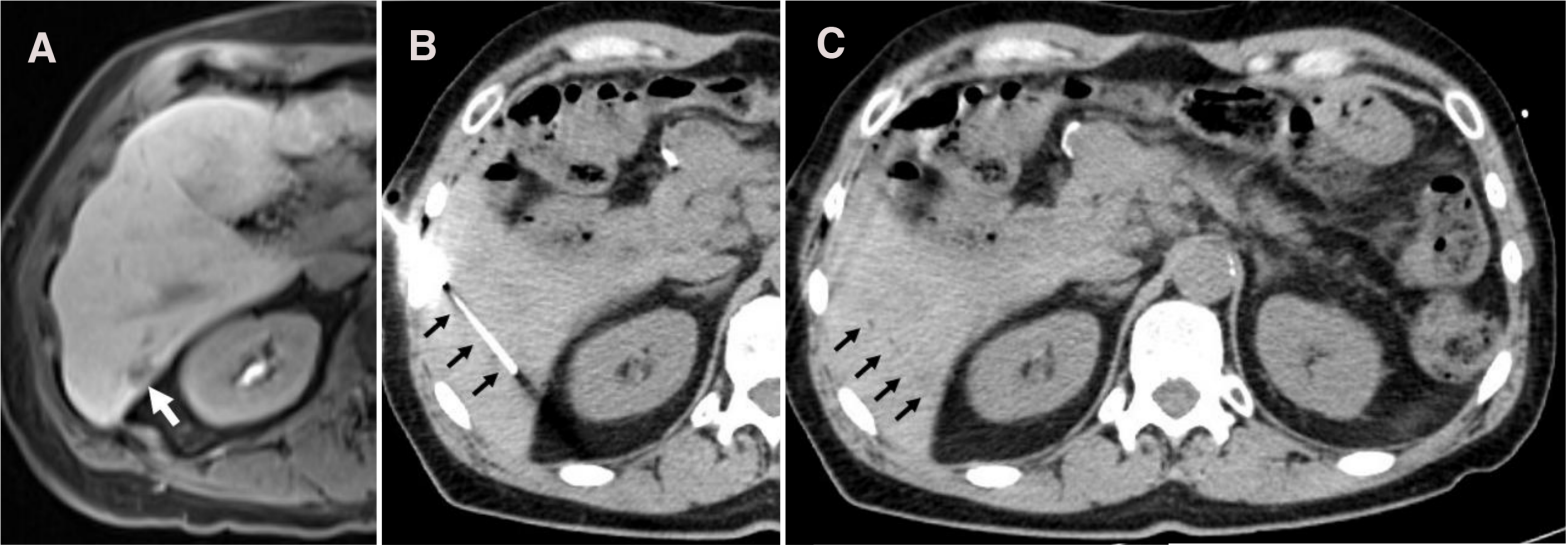
Case #5, Panel **(A)** Liver mass seen by delayed phase T1 post-contrast MRI (white arrow). Panel **(B)** Radiopaque needle seen during biopsy under CT guidance (black arrows). Panel **(C)** Thin line of gas (black arrows) seen on CT following electrocautery with the SinglePass device.

A 65-year-old male underwent an outpatient CNB procedure due to the finding of a liver lesion in the right lobe of his liver (**Figure 4A**). The patient had a history of adenocarcinoma of ampulla of Vater status post-Whipple procedure and prior chemotherapy. The patient was not taking antiplatelet or anticoagulant medications, and all pre-procedure laboratory testing was within normal limits.

The biopsy procedure was followed by SinglePass device electrocautery, both under CT guidance with four core samples obtained. Intraprocedural CT images show appropriate needle access to the lesion (**Figure 4B**). Post-procedural CT images show no evidence of bleeding complication; furthermore, faint linear gas can be seen along the access tract, felt to be secondary to cautery effect (**Figure 4C**). The patient was discharged 4 hours post-procedure following CT confirmation of hemostasis. There were no complications reported in the 30 days following the procedure.

The pathology found moderately differentiated adenocarcinoma morphologically compatible with that from the prior resection specimen, consistent with metastatic disease. The patient went on to receive FOLFIRINOX and, thereafter, elected to enter a clinical trial.

## Discussion

Bleeding complications in percutaneous needle biopsies are an ongoing concern despite advances in equipment and techniques. Despite bleeding risks, there is an increasing interest in obtaining robust core biopsy specimens in the age of novel testing including next generation sequencing for characterization of malignancy. Liver mass biopsies are frequently required for diagnosis and staging of malignancy yet carry a risk of bleeding. As such, many centers, including ours, routinely perform liver mass biopsies using 19/20-gauge biopsy systems. These systems yield much smaller tissue samples, require more needle passes to obtain adequate tissue, and can, in fact, be counterproductive (15).

The SinglePass device is a novel electrocautery device that has demonstrated effectiveness in quickly and durably obtaining needle tract hemostasis during this early case series of liver mass CNB procedures as well as in two non-oncologic case series. Furthermore, due to the ability of the SinglePass device to close 17-gauge needle tracts as part of 17/18-gauge biopsy systems, this enhanced our ability to obtain abundant and high-quality tissue. In each case, not only were there no bleeding complications, but there was adequate tissue for diagnosis. This report of limited experience is consistent with larger series that employ needle tract plugging techniques to minimize the incidence of major bleeding events (16–18). While the addition of the SinglePass device to CNB procedures adds cost, the SinglePass device is more easily controlled than injectables thus reducing case-to-care variability. Additionally, its rapid learning curve allows seamless incorporation into the standard Interventional Radiology procedural workflow within relatively few cases of experience.

Routine SinglePass device use has the potential to overcome bleeding unpredictability thereby becoming a valuable tool in minimizing potentially life-threatening hemorrhagic complications. Furthermore, the SinglePass device may enable interventional radiologists to more routinely obtain larger-gauge biopsy samples due to the ability to obtain active needle tract cautery after biopsy completion.

Given the potential for perceived bias due to industry involvement and the absence of independent outcome adjudication in this case series, the need for independent, non-industry-funded study is needed, Larger-scale, comparative evaluations to determine the extent of the SinglePass device’s ability to reduce bleeding events relative to established needle tract hemostasis techniques are warranted.

## Data Availability

The raw data supporting the conclusions of this article will be made available by the authors, without undue reservation.
